# Detection of *Bartonella schoenbuchensis* and a novel sigmavirus within the microbiome of deer keds (*Lipoptena cervi*) from the United Kingdom

**DOI:** 10.1186/s13071-025-07208-w

**Published:** 2026-01-25

**Authors:** Ben P. Jones, Denise C. Wawman, Nicholas Johnson

**Affiliations:** 1 Vector-Borne Disease Workgroup, Virology Department, Animal and Plant Health Agency, Woodham Lane, Addlestone, KT15 3NB UK; 2 Edward Grey Institute of Field Ornithology, Department of Biology, University of Oxford, South Parks Road, Oxford, OX1 3RB UK; 3 Faculty of Health and Medical Sciences, University of Surrey, Guildford, GU2 7XH UK

**Keywords:** Deer keds, Microbiome, Endosymbionts, Sigmavirus, Hippoboscidae

## Abstract

**Background:**

*Lipoptena cervi* is a member of the Hippoboscidae family of insects and is a hematophagous ectoparasite of cervid species, commonly referred to as the deer ked. *Lipoptena cervi* has a wide geographical distribution and can be found from North America through Europe into East Asia. Deer keds occasionally bite humans and domestic animals and might act as disease vectors. The microbiome associated with this species of biting insect has not been investigated.

**Methods:**

Mass sequencing of both DNA and RNA extracted from *L. cervi* specimens collected from two locations in southern England was conducted to characterise the complete microbiome consisting of bacterial, viral and eukaryotic species. Three specimens were collected after landing on humans in Somerset, and three specimens were collected from European roe deer (*Capreolus capreolus*) in Oxfordshire. Bioinformatic analysis investigated the host and microbial composition of each specimen.

**Results:**

Near-complete mitochondrial genomes were assembled from all six specimens confirming morphological speciation as *L. cervi*. Bacterial endosymbionts were the most dominant organisms identified with *Candidatus* Arsenophonus lipoptenae being most abundant. In specimens that had fed on deer, the pathogen *Bartonella schoenbuchensis* was detected. A novel sigmavirus was also detected in five samples, four of which yielded near-complete genomes. The closest relative of this virus was a sigmavirus found in a sheep ked (*Melophagus ovinus*) sampled in the Russian Federation.

**Conclusions:**

The data from this study will allow for a better baseline understanding of the microbiome of *L. cervi* and provide evidence for their role as vectors of zoonotic pathogens.

**Graphical Abstract:**

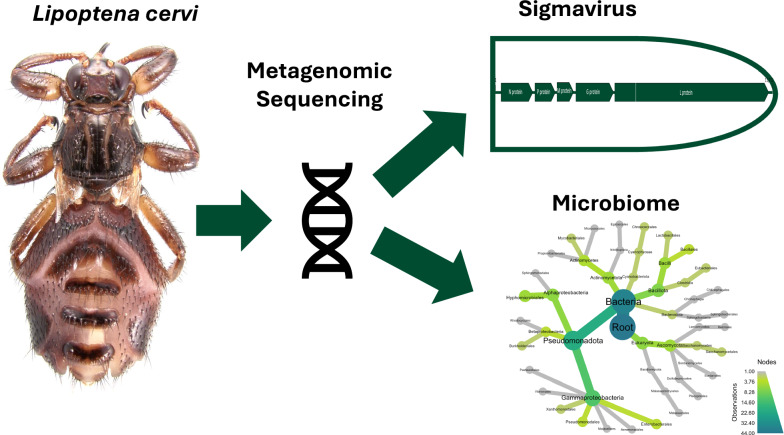

**Supplementary Information:**

The online version contains supplementary material available at 10.1186/s13071-025-07208-w.

## Background

The deer ked (*Lipoptena cervi*) is a widespread hematophagous ectoparasitic fly of cervids found across temperate regions of the northern hemisphere. The species has been reported in Europe, North America and Asia, with detection as far east as Korea [1]. They belong to the family Hippoboscidae (louse flies and keds) and are within the subfamily Lipopteninae, which includes three genera, *Lipoptena*, *Melophagus* and *Neolipotena*. Unlike the sheep ked (*Melophagus ovinus*), the adult forms of *L. cervi* are winged, allowing them to fly short distances to locate a vertebrate host. Once on the host, the wings are discarded, and both male and females take a blood meal. Copulation occurs on the host with the females producing live prepupae that pupate immediately. These fall to the ground, emerging later in the summer as winged adults. Although primarily an ectoparasite of cervids, of which there are six species in the British Isles, deer keds are reported as a biting nuisance for humans, occasionally resulting in a persistent dermatitis [2, 3]. Deer keds have also been reported on companion animals, particularly dogs [4, 5].

Deer keds have been considered potential vectors of pathogens including transmission to humans [6]. The strongest evidence for persistent infection in keds and likely transmission to vertebrate hosts is for the bacterium *Bartonella schoenbuchensis*. High levels of bacteraemia with *B. schoenbuchensis* were reported in European roe deer (*Capreolus capreolus*) in Germany [7]. The bacterium has been visualised within the midgut of *L. cervi* removed from deer, and vertical transmission from adult females to larvae has been demonstrated [8, 9]. Several other bacterial pathogens have been detected in *L. cervi*, including *Anaplasma phagocytophilum*, *Borrelia burgdorferi*, *Rickettsia* spp. and *Acinetobacter* spp. [10, 11]. Another group of potential pathogens associated with *L. cervi* are the trypanosomes of deer. These were first identified in the mid- and hind-gut of deer keds in Germany and later in North America [12, 13].

Whilst metagenomic analysis to elucidate the biomes within many blood-feeding arthropods, such as ticks and mosquitoes, is common, limited research of this kind has been performed in Hippoboscidae [14–16]. Most studies focus only on the bacterial microbiome via analysis of 16S rRNA sequences, missing key information on viral and eukaryotic biomes, which may include potential pathogens [17–20]. There is abundant evidence that the deer ked *L. cervi* is host to several microorganisms, some of which may be pathogenic for humans. To gain a comprehensive overview of the full microbiome of this species (inclusive of bacteria, viruses and eukaryotes), we investigated intact adults using mass sequencing and bioinformatic analysis of *L. cervi* specimens obtained from humans and roe deer in the UK to assess the risk of disease transmission.

## Methods

### Sample collection

**Fig. 1:**
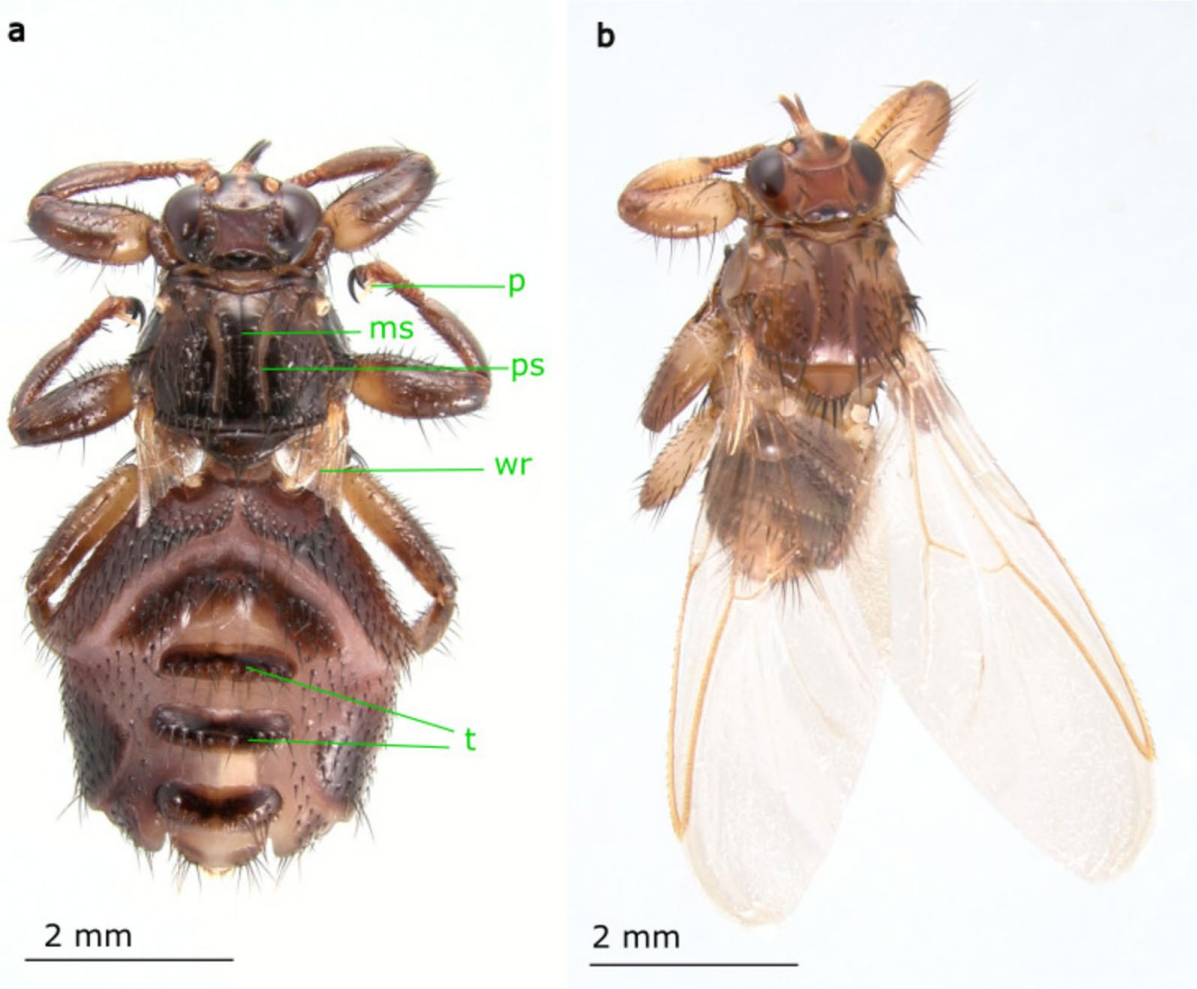
Images of *Lipoptena cervi* female morphology. (**a**) Dealate fly showing the remains of the wings (wr), with the abdominal tergites (t) clearly visible following a blood meal. This species can be distinguished from other species in the genus by its size relative to other *Lipoptena* species and by a range of morphological features, including the presence of fully developed pulvilli (p), a medionotal or longitudinal suture (ms) and prescutellar sutures (ps) on the mesoscutum, the arrangement of bristles on the dorsal thorax, the shape of the female terminalia and the aedeagus in males (not clearly visible in either photograph); **(b**) unfed alate (winged) fly. The images are representative of *L. cervi* morphology and are not of the individuals analysed in this study

As part of a wider study to investigate the diversity and ecology of louse flies in the UK, six *L. cervi* samples were collected from two locations (Table 1). Three individuals were collected after landing on humans in the Somerset County prior to feeding. Species identification was made based on morphology (Fig. 1) [21]. However, the sex of these flies could not be determined definitively. Three dealate individuals, two females and one male, were sampled from recently culled roe deer in Oxfordshire and had taken a blood meal. The flies were placed in RNAlater (Thermo Fisher Scientific, Crawley, UK) for later analyses.

**Table 1:** Metadata on *Lipoptena cervi* specimens and a summary of sequences aligned to Hippoboscidae host for both DNA and RNA samples

Sample ref	Sex (M/F/U)^a^	Fed (Y/N)	Location (county)	Source (human/deer)	No. of filtered reads (M) (DNA sample)	DNA % aligned to host	No. of filtered reads (M) (RNA sample)	RNA % aligned to host
LC89	U	N	Somerset	Human	20.8	4.00%	19.9	96.00%
LC90	U	N	Somerset	Human	17	3.40%	19.9	96.10%
LC91	U	N	Somerset	Human	29	3.80%	18.2	96.00%
LC102	F	Y	Oxfordshire	Roe deer	19.2	4.30%	18.2	95.50%
LC103	M	Y	Oxfordshire	Roe deer	17.2	4.50%	7.3	95.10%
LC14	F	Y	Oxfordshire	Roe deer	19.8	4.30%	19.6	94.20%

^a^Male (M), female (F), unknown (U)

### Nucleic acid preparation

Separate DNA and RNA were isolated from six individual deer keds using the QIAgen AllPrep DNA/RNA MiniKit (Qiagen, Manchester, UK). Briefly, adult flies were surface sterilised with 500 µl 5% sodium hypochlorite, vortexed intermittently for 2 min and then washed twice with 1000 µl phosphate-buffered saline. The flies were homogenised in 300 µl RTL buffer using a single 5-mm steel bead in a TissueLyserII (Qiagen) for 5 min at 30 Hz. Nucleic acid was prepared following the manufacturer’s instructions with DNA eluted into 60 µl buffer EB and RNA eluted into 50 µl RNase-free water. Sequencing libraries were prepared using Nexetra XT kits (Illumina, Cambridge, UK) and sequencing was performed using a NextSeq 550 sequencer (Illumina, Cambridge, UK) to generate 2 × 150 base pair-end reads.

### Quality checks and host removal

Raw reads were trimmed using FastP (v0.23.4) [22]. A host reference database comprising the complete reference genomes of all Hippoboscidae sequences from the National Centre for Biotechnology Information (NCBI), as of 30 April 2024, consisting of *Melophagus ovinus*: GCA_023089585.1; *Crataerina pallida*: GCA_949710015.1; *Ornithomya chloropus*: GCA_963971445.1; *Ornithomya fringillina*: GCA_963978525.1, was created within Bowtie2 (v2.5.2)[23]. Reads originating from the host were separated from non-host by mapping the filtered reads to a host reference database using Bowtie2. Reports from both tools were used to create a combined QC report using MultiQC (v1.19) [24] (Table 1).

### Mitogenome phylogenetics

MEGAHIT (v1.2.9) [25] was used to assemble contigs de novo. A reference database was created consisting of the mitochondrial genomes of Hippoboscidae and was used to perform a BLAST (v2.5.0) [26] search of de novo assembled contigs to identify conserved regions within the mitochondrion. The contigs identified via this process were then mapped back to the reference *Lipoptena* sp. sequence (MT679542.1) using Geneious Prime (v 2024.0.5. Biomatters Ltd.) and the consensus was extracted to create the mitogenome of each sample. The mitogenome with the fewest ambiguities was subsequently used as a reference genome for reference-based alignment using Bowtie2. To generate a phylogeny for the genus *Lipoptena*, a 502-bp segment of the cytochrome c oxidase subunit I (*COI*) gene was extracted and aligned with other sequences from *Lipoptena*. The alignment was created in Geneious Prime using the ClustalW algorithm. Phylogenies were created using MrBayes (v 3.2.7a) [27] with 1,000,000 generations of Markov chain Monte Carlo simulations under the GTR + G + I model. A second phylogeny was created utilising the complete coding region of the mitogenomes with all other Hippoboscidae mitogenomes. This second phylogeny was created using MrBayes with 1,000,000 generations of Markov chain Monte Carlo simulations under the GTR + G + I model.

### Taxonomic classification of microbial sequence reads

The host-filtered reads were used for taxonomic classification with Kraken2 (v2.1.3) [28] using the prebuilt PlusPF index database (last updated 12 January 2024). A confidence threshold in Kraken2 was set at 0.3 and a minimum of three hit-groups was required before a sequence could be classified. Bracken (v2.9) [29] was then used to transform the Kraken2 results into read abundance and filter out any taxon for which there were < 10 hits. The results from Bracken were converted to biom format using kraken-biom (v1.2.0) [30] and analysed in RStudio (v2023.12.1) [31] using R (v4.4.0) [32] and package phyloseq (v1.48.0) [33] to remove any human reads, create relative abundance plots and perform diversity analysis. The R package, Metacoder (v0.3.7) [34], was used to create a taxonomic heat tree to display the diversity of taxa found within the dataset. To identify dissimilarity between the populations of *L. cervi*, principal coordinate analyses (PCoA) using the Bray-Curtis dissimilarity statistics were performed in R using phyloseq.

### Assembly and pathogen detection

Initial de novo assembly was performed using MEGAHIT and the contigs produced were used to identify viruses using ViralVerify (v1.1) [35] and ViralComplete [35]. The outputs of these programmes then underwent BLAST screening using the NCBI “viruses_nt” database (available at: https://ftp.ncbi.nlm.nih.gov/blast/db last updated 31 January 2024) to identify the viruses picked up by the pathogen detection software. Viruses identified by these methods, with a minimum alignment length of 500 bp, were taken as true positives and were run through the online BLAST server using the core nucleotide database.

For any viruses identified, the closest matching reference sequence was downloaded from NCBI. All samples were then screened using Bowtie2 to map reads to the virus reference sequence. These reference-mapped sequences were then further analysed. For each viral sequence identified, an alignment of the translated amino acid sequence of the RNA-dependant RNA polymerase (RdRp) was created in Geneious Prime using the ClustalW algorithm. Phylogenies were created using MrBayes with 1,000,000 generations of Markov chain Monte Carlo simulations under the VT model. The best model for each dataset was predicted using Modeltest-ng (v0.1.7.) [36]. The resulting Bayesian phylogenies were visualised in Figtree (v1.4.4.) [37].

### Pathogen and endosymbiont sequence detection

To gain a deeper insight into the detection of potential pathogens and endosymbionts identified in the taxonomic assignment, sequences were isolated using reference-guided alignment with Bowtie2. For *Bartonella schoenbuchensis*, citrate synthase (*gltA*) (FN645507.1) was used, and for *Candidatus* Arsenophonus lipoptenae, 16S ribosomal RNA (CP013920.1) was implemented, as these markers can identify each species and allow the best comparisons. Alignments were created in Geneious Prime using the ClustalW algorithm. Phylogenies were created using MrBayes with 100,000,000 generations of Markov chain Monte Carlo simulations under the HKY + G model. In addition, multi-locus sequence typing (MLST) was performed for *B. schoenbuchensis* using the MLST sequences proposed by Vogt et al. [38]. Target sequences were isolated using reference-guided alignment with Bowtie2. Sequences from this study were compared with the sequences obtained from the supplementary material of Vogt et al. [38]. A Bayesian phylogeny was created for the concatenated MLST sequences using MrBayes with 1,000,000 generations of Markov chain Monte Carlo simulations using the HKY + I model.

## Results

### Identification and sequencing of deer keds

In this study we performed metagenomic analysis on both DNA and RNA extracted from six deer keds collected in the UK. The deer keds were morphologically identified as *L. cervi* (Fig. 1). These samples consisted of three keds collected after landing on humans in Somerset and three keds collected from roe deer in Oxfordshire (Table 1). Only the keds collected from deer had fed on their host. After filtering the raw data, each sample had an average of 18.84 million (M) reads. For DNA samples an average of 4.05% of reads aligned to the Hippoboscidae host database and were removed, whereas for the RNA samples an average of 95.48% of reads mapped back to the host database (Table 1).

### Mitogenomic analysis to confirm morphological identifications

Mitochondrial genomes could not be obtained via reference-based alignment because of the lack of a reference mitogenome for *L. cervi*, with the closest sequence being that of a “*Lipoptena* sp.” (accession no. MT679542.1), which resulted in a mitogenome with many gaps and ambiguities. Therefore, de novo assembled contigs from MEGAHIT were used to blast against this *Lipoptena* sp. reference (MT679542.1) to identify large contigs containing conserved regions within the mitochondrion. The contigs identified via this process were then mapped back to the reference sequence and the consensus extracted to create the mitogenome of each sample. The mitogenome with the fewest ambiguities was subsequently used as a reference genome for reference-based alignment using Bowtie2.

**Fig. 2:**
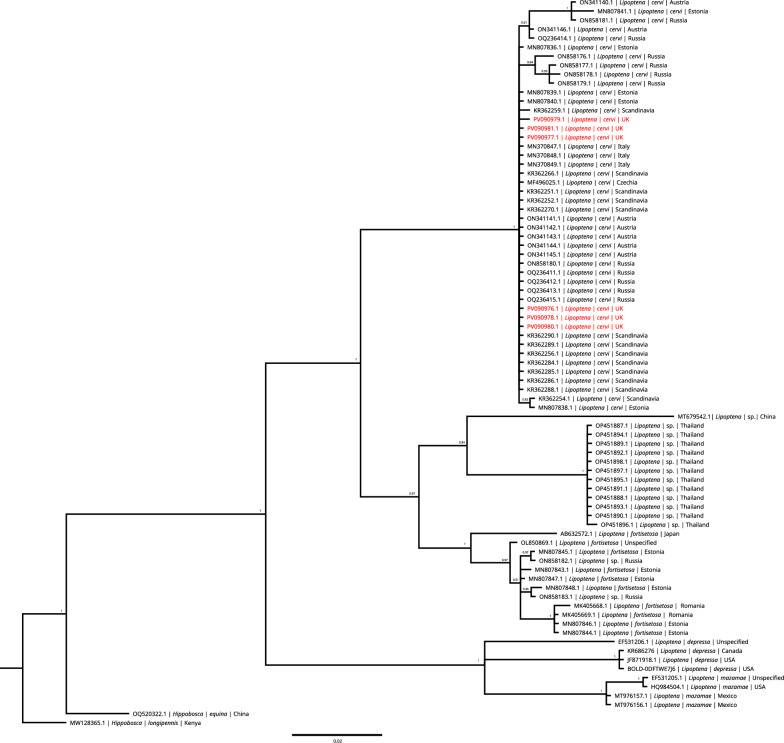
Species confirmation of *Lipoptena cervi* sequences derived from deer keds sampled in the UK. Bayesian phylogeny of the genus *Lipoptena* based on an alignment of a 502 base pair sequence of the *COI* gene. Each node is labelled with Bayesian posterior probability. Sequences obtained in this study are highlighted in red and cluster within the *L. cervi* group

The *L. cervi* samples used in this study produced six partial mitogenomes containing the complete coding region, but with a truncated control region, and were submitted to Genbank (accession nos. PV090976–PV090981). A 502-bp region of the *COI* gene, a sequence commonly used for species identification, was aligned with available sequences from *Lipoptena* species to generate a phylogeny (Fig. 2). *Hippobosca* spp. sequences were used as an outgroup. The phylogenetic analysis confirmed that all samples were *L. cervi* with high posterior probability. The *Lipoptena* sp. sequence initially used as a reference clustered with other *Lipoptena* sp. sequences from Asia, a sister clade to *Lipoptena fortisetosa* sequences from Eastern Europe/Russia. The phylogeny created using the complete mitogenomes only contained one other *Lipoptena* sequence, which was clustered most closely with the isolates from this study but did not help resolve the sequences to a species level (Additional file 1).

### Characterising the microbiome of deer keds

**Fig. 3:**
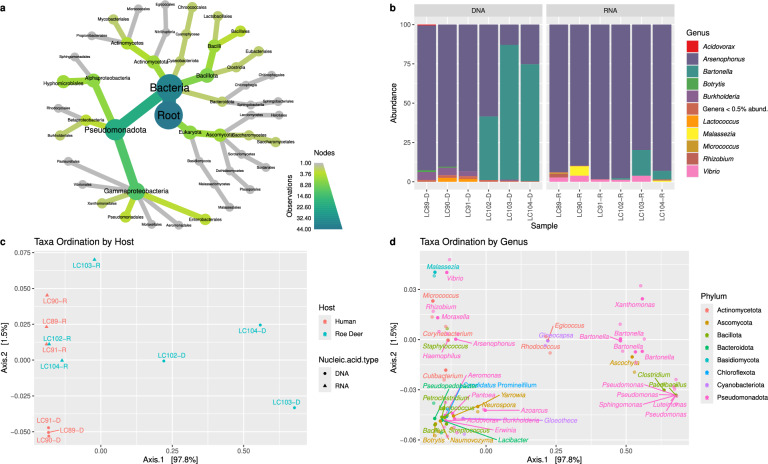
Microbiome of *Lipoptena cervi*. **a** The taxonomic heat tree shows the composition of organisms found within the whole dataset. The colour of the nodes indicates the number of organisms taxonomically assigned to the group. **b** Bar plot showing the relative abundance of bacterial genera identified in each sample. **c** PCoA analysis by host and nucleic acid type. The colour of each sample represents the host species from which the ked was found and the shape represents the nucleic acid type analysed. **d** PCoA analysis by phylum. Each point on the PCoA represents an organism identified within the dataset labelled at the genus level and coloured by the phylum. The distribution of taxa within this plot can be compared with the PCoA of the samples to illustrate which taxa play a role in differentiating samples

Characterisation of the microbiome of *L. cervi* identified a range of microorganisms. For all samples most non-host reads were derived from bacterial species with an average 99.57% and 98.86% of reads from DNA and RNA samples, respectively. The overall composition of the microbiome in all samples as predicted by taxonomic assignment is shown in the taxonomic heat tree (Fig. 3a) and the composition of organisms from individual samples is given in the relative abundance plots (Fig. 3b).

*Arsenophonus* endosymbionts were detected in all samples and were the dominant organism found in all RNA samples. Both RNA and DNA samples of the three flies from Somerset were collected following contact with humans. In contrast, the samples from Oxfordshire, collected from roe deer, had a much lower abundance of *Arsenophonus* sequence reads in their DNA. In these samples a high level of *Bartonella* sequences was identified in the DNA sample, which was also observed to a lesser degree in the RNA sample. *Bartonella* sequences were only observed in the samples from deer in Oxfordshire and were not found in the samples from humans in Somerset.

Other bacterial genera detected in the samples with an abundance of ≥ 0.5% included *Burkholderia*, *Lactococcus*, *Micrococcus*, *Rhizobium* and *Vibrio*. The fungal genera *Botrys* and *Malassezia* were also detected at this level.

Principal coordinate analysis (PCoA) was performed to identify relationships between the microbiomes of samples. Principal coordinate analyses plots were produced based on both individual samples and the taxa composition of the microbiome itself (Fig. 3c, d, respectively). The PCoA focused on individual samples showed a difference between DNA and RNA samples. The RNA samples from all six *L. cervi* specimens grouped relatively closely together, indicating that the microbiome composition based on RNA is similar between all specimens. Larger differences were observed between the DNA microbiomes. Not only were they separate from the RNA samples, but the plots also indicated that the samples from the two different locations were different from each other. The three DNA samples from *L. cervi* collected from humans in Somerset clustered tightly, indicating a conserved microbiome. The three DNA samples from *L. cervi* were collected from roe deer separated by a great distance from the other samples and showed relatively large separation from each other, indicating a more diverse microbiome composition (Fig. 3c). Comparing the PCoA based on taxa showed the distribution of each taxon based on its abundance within the dataset (Fig. 3d). The *Bartonella* points all cluster closely together, indicating that they have similar distribution patterns. Comparing this to Fig. 3c, we observed that the three samples containing high levels of *Bartonella* also clustered away from the rest of the samples. This indicated that the presence of *Bartonella* could be one of the factors separating the DNA samples collected from deer.

### Identification of specific bacterial species within deer keds

**Fig. 4:**
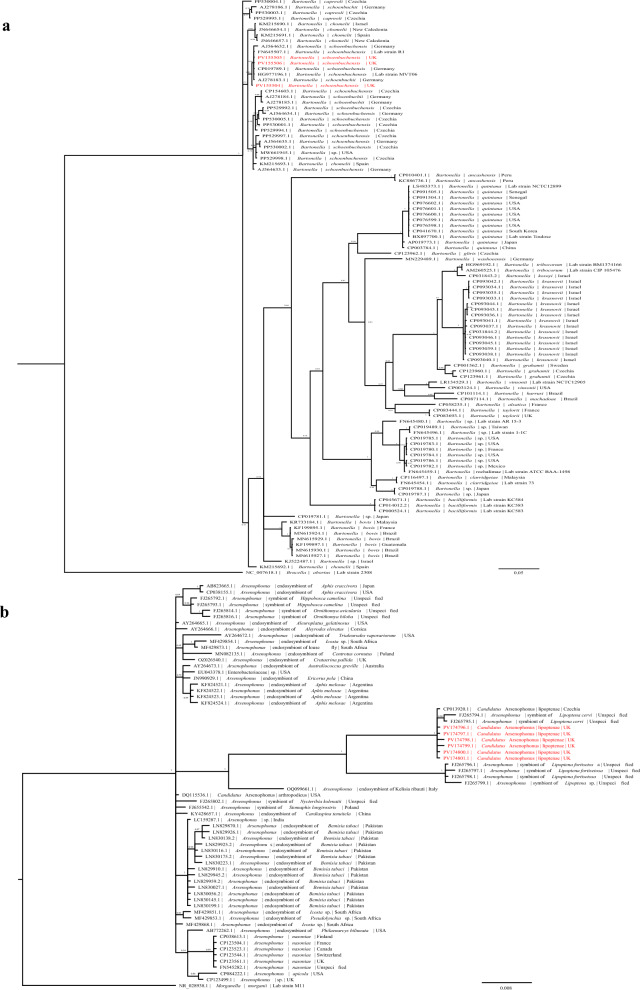
Endosymbiont phylogenies. **a** The phylogeny was created using a 659-bp conserved region of the *gltA* gene from other *Bartonella* isolates, with *Brucella abortus* (another member of the order Hyphomicrobiales) used as an outgroup. This places the deer ked sequences within the *Bartonella schoenbuchensis* clade. **b** A phylogeny was created using a 975-bp conserved region of the 16S rRNA gene from other *Arsenophonus* isolates from invertebrates with *Morganella morganii* (another member of the family Morganellaceae) used as an outgroup. The phylogenies show that the deer ked sequences cluster closely to *Arsenophus* sequences found in *Lipoptena* sp. Each node is labelled with Bayesian posterior probability. Sequences obtained in this study are highlighted in red

Near-complete sequences of the *Bartonella* *gltA* gene were derived from the DNA of two of the three flies from deer (LC102-D and LC103-D), and the complete sequence was identified from the third (LC104-D) (accession nos. PV155504–PV155506). Although *Bartonella* was identified in the RNA samples of these isolates in the global microbiome analysis, significant *gltA* gene sequences could not be retrieved in the RNA samples from these isolates. Phylogenetic analysis showed the *Bartonella* sequences obtained in this study fell within a cluster of *B. schoenbuchensis* sequences, an organism known to infect deer keds (Fig. 4a). Compared to concatenated MLST sequences from a previous study, the MLST of *Bartonella* from the isolates in this study clustered with *B. schoenbuchensis* (Additional file 2) [38]. Sequences obtained for each gene in the MLST were deposited in NCBI (accession nos. PV155504–PV155506, PX206320–PX206331 and PX208520–PX208522).

The complete 16S ribosomal RNA of *Arsenophonus*, the most abundant bacterial species detected, was successfully determined from five of the six DNA samples, with a near-complete 16S ribosomal RNA sequence found in the remaining specimen (accession nos. PV174796–PV174801). When phylogenetically compared to *Arsenophonus* endosymbionts from other invertebrates, the sequences isolated in this study clustered with *Ca.* A. lipoptenae sequences (Fig. 4b).

### The virome of *L. cervi*: characterisation of a novel sigmavirus

**Fig. 5:**
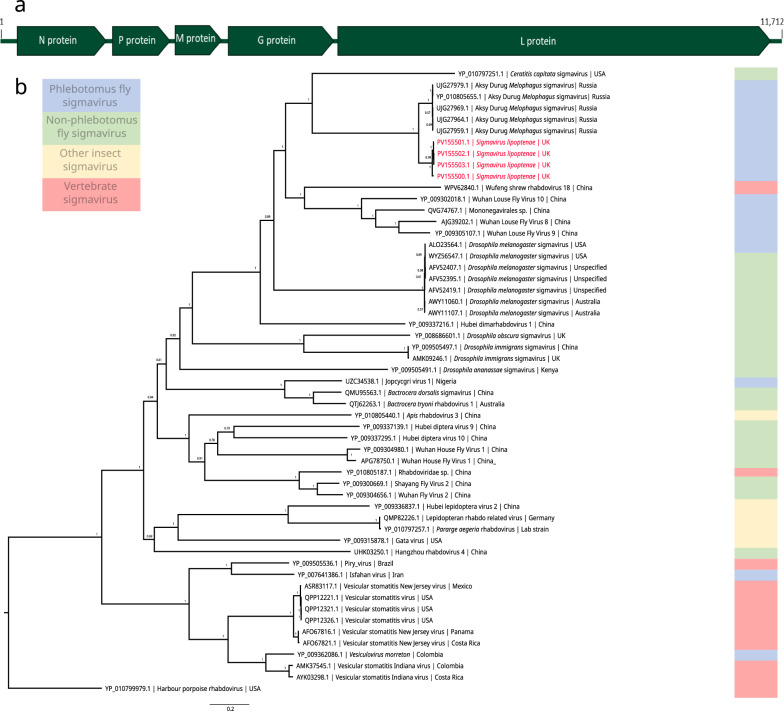
Detection of a sigmavirus in *Lipoptena cervi*. **a** Schematic showing typical Sigmavirus genome (N-P-M-G-L) with annotations from deer ked sigmavirus. **b** Phylogeny showing the relationship of the sigmavirus identified in this study to other sigmaviruses based on RdRp amino acid sequence. Each node is labelled with Bayesian posterior probability. Sequences obtained in this study are highlighted in red

Overall, few viruses were identified within the small number of deer keds tested. One virus was found with near-complete genomes in multiple samples and two partial sequences of other viruses were identified. The virus detection software employed was able to identify and characterise a novel sigmavirus in *L. cervi*, designated *Sigmavirus lipoptenae*. Near-complete genomes were isolated from four samples, LC89-R, LC102-R, LC103-R and LC104-R (accession nos. PV155500–PV155503) and small fragments were identified in one additional sample (LC90-R). The genomic structure of this virus was typical of Rhabdovirus genomes with the coding sequence order of nucleoprotein (N), phosphoprotein (P), matrix (M), glycoprotein (G) and RNA-dependent RNA polymerase (L). The genome length was approximately 11.8-k base pairs (Fig. 5a). No other coding sequences were identified. Phylogenetic analysis of the genomic sequences derived from *L. cervi* demonstrated that the virus with the highest sequence identity was Aksy-Durug Melophagus sigmavirus (ADMSV), a virus identified in *Melophagus ovinus* flies from Russia. The viruses share an average of 98.29% identity within the *L. cervi* group and an average of 87.96% with ADMSV isolates in the RdRp amino acid sequence (Table 2). The phylogeny shows that these sigmaviruses cluster with viruses isolated from other phlebotomus flies and vertebrates (Fig. 5b).

**Table 2:** Amino acid similarities (%) between the sigmavirus found in this study compared to other sigmaviruses based on the pairwise identity of the RdRp protein

	UJG27979.1 ADMSV	YP_010805655.1 ADMSV	UJG27969.1 ADMSV	UJG27964.1 ADMSV	UJG27959.1 ADMSV	LC102-R_Sigmavirus	LC89-R_Sigmavirus	LC103-R_Sigmavirus	LC104-R_Sigmavirus
UJG27979.1 ADMSV		99.953	99.801	99.72	99.814	86.91	87.925	88.112	88.07
YP_010805655.1 ADMSV	99.953		99.752	99.674	99.767	86.864	87.879	88.065	88.023
UJG27969.1 ADMSV	99.801	99.752		99.801	99.901	87.924	88.922	89.121	89.076
UJG27964.1 ADMSV	99.72	99.674	99.801		99.907	86.91	87.925	88.112	88.07
UJG27959.1 ADMSV	99.814	99.767	99.901	99.907		86.957	87.972	88.159	88.116
LC102-R_Sigmavirus	86.91	86.864	87.924	86.91	86.957		97.074	97.773	97.684
LC89-R_Sigmavirus	87.925	87.879	88.922	87.925	87.972	97.074		98.695	98.606
LC103-R_Sigmavirus	88.112	88.065	89.121	88.112	88.159	97.773	98.695		99.911
LC104-R_Sigmavirus	88.07	88.023	89.076	88.07	88.116	97.684	98.606	99.911	

In addition to the near-complete sigmavirus, two partial sequences were identified. In one DNA sample, LC102-D, a 4556-bp sequence was identified that had the highest sequence identity to the VP2 segment of Shelly Beach virus isolated from ticks in Australia [39]. In RNA sample LC104-R, a 3092-bp sequence was identified that had the highest similarity to several sobemo-like viruses from batflies and other blood-feeding insects in China [16, 40]. This sobemo-like sequence contained two hypothetical proteins (Table 3).

**Table 3:** The closest matching viruses to the partial viral sequences identified in this study based on BLAST and including host species in which the reference virus was found

Sample	Virus	Cover	Identity	Virus family	Invertebrate host	Accession
LC102-D	Shelly Beach virus VP2	46%	75.42%	Reoviridae	*Ixodes holocyclus* (Australia)	AYP67578.1
LC104-R	Yunan sobemo-like virus hypothetical protein 1	38%	73.50%	Solemoviridae	Nycteribiidae (China)	QVG74747.1
	Yunan sobemo-like virus hypothetical protein 2	53%	51.64%	Solemoviridae	Nycteribiidae (China)	QVG74744.1

## Discussion

Keds are increasingly being recognised as nuisance biting insects with the potential to vector pathogenic organisms [6]. Further data on the microbiome associated with geographically dispersed species such as *L. cervi* will increase understanding of this neglected ectoparasite. This study enabled the assembly of three high-quality, near-complete mitochondrial genomes of *L. cervi* for the first time. The closest available mitochondrial genome is that of a “*Lipoptena sp.*”, derived from a specimen sampled in China, which proved too divergent to allow accurate mapping of reads from *L. cervi*. The phylogeny based on a partial *COI* gene sequence shows that the published *Lipoptena* sp. from China are distinct from *L. cervi* and form a separate clade more closely related to *Lipoptena fortisetosa*.

The microbiome of all samples was dominated by *Ca.* A. lipoptenae. This is representative of its role as a key endosymbiont in providing nutritional support to *L. cervi*, which is required because of its exclusively haematophagic diet. *Candidatus* Arsenophonus lipoptenae have been visualised within specialised cells in the gut wall of *L. cervi* known as bacteriocytes [41]. Like many other endosymbionts of blood-feeding insects, *Ca.* A. lipoptena is believed to play a crucial role in the synthesis of nutrients such as B vitamins, which are deficient in the blood-meal diet of *L. cervi* [41]. However, in *Ca*. A. lipoptenae and other well-characterised species of *Arsenophonus*, whilst the pathways for many B vitamins are present, the pathway for vitamin pantothenate (B5) is missing [41–44]. It has been suggested that other bacteria, such as *Bartonella* sp. and *Sodalis* spp., can act as symbionts to produce this in insects [42, 45].

*Bartonella* spp. are known pathogens that can lead to diseases such as cat-scratch disease and trench fever. *Bartonella schoenbuchensis* has been associated with Hippoboscid flies [8, 18, 38, 46–48] and has been detected in several of its ruminant hosts [7, 18, 47, 49]. *Bartonella schoenbuchensis* infection in humans occasionally causes deer ked dermatitis, which is symptomatically similar to cat-scratch disease (caused by *Bartonella henselae*) [8, 50]. In this study *B. schoenbuchensis* was only detected in flies that had previously fed on roe deer. This suggests that the *L. cervi* specimens acquired the bacteria from the deer on which they were feeding and that the endosymbiont was associated with the blood meal or was stimulated by blood ingestion. However, previous studies have suggested that *B. schoenbuchensis* is vertically transmitted from mother to offspring. As the samples from Somerset had not fed, we could not determine whether feeding is critical to acquiring *B. schoenbuchensis* or if there are geographical differences in the distribution of this bacterial species in southern England.

To the best of our knowledge, this is the first description of a sigmavirus within *L. cervi*. The closest relative of this novel sigmavirus has been found in other sheep keds in Russia, indicating that it is likely a ked-specific virus. Sigmaviruses of fruit flies transmit vertically and therefore do not require transmission to a vertebrate host, although host-switching is believed to occur [51–53]. This virus was detected in five of six samples, supporting an intimate association with *L. cervi*. Whether this is true for other insect-associated sigmaviruses is yet to be determined. The virus identified in this study clusters with other sigmaviruses from Hippoboscidae but also with a virus found in a shrew in China. Aksy-Durug Melophagus sigmavirus was originally shown to be able to be passaged multiple times through porcine embryo kidney cell lines; it is therefore hypothesised that this virus also has the potential to infect vertebrates [14]. However, further research is required to understand the risk of sigmavirus infection in vertebrates.

The sequencing of both RNA and DNA was performed to test whether the same profiles would be detected within a single sample. This methodology introduces the possibility of biases between sample types via several causes, most notably because RNA is less stable than DNA and additional steps are needed in library preparation. Both RNA and DNA were extracted using filter column kits at the same time, which should reduce the bias based on extraction methodologies. Based on the quality results seen in Table 1, we showed that the number of high-quality reads for both DNA and RNA samples is relatively consistent. However, there is a large difference in the amount of data that aligns with the host genome and is therefore removed prior to microbiome analysis. This results in fewer reads being analysed for RNA. The results demonstrated that, overall, the species identified within the samples are mostly similar, but the proportion of each varies. This is most obvious for *Bartonella* where it represents a large proportion of the DNA of three samples but a much lower proportion of RNA. This could indicate that in these samples, although there are many *Bartonella* cells, they are not transcriptionally active.

The samples positive for *Bartonella* were the keds that had fed on deer blood; therefore, one explanation may be that the bacteria identified were present within the blood meal, perhaps with a high load, but not all were actively replicating. To test this theory, a larger selection of both fed and unfed individuals would need to be analysed. Notably, *Bartonella* in general is known to be a slow-growing bacterium which could be affecting its transcriptional activity relative to the other main organism found in these samples, *Ca.* A. lipoptena [54].

The findings of organisms such as the yeast *Malassezia* and the bacterium *Vibro* only in the RNA samples are interesting, and there could be several reasons for this finding. In contrast to *Bartonella*, there could be a small number of individual organisms, so they are missed in the DNA sampling, but they could be highly transcriptionally active, which is why we see them in the RNA. Further screening is needed to confirm these findings. *Malassezia* are a genus of potentially pathogenic yeast commonly associated with mammal skin and may have been passed onto the ked when it landed on a mammalian host; however, as the samples were surface sterilised, the organisms would have to have been internalised into the ked to be detected. The *Vibrio* genus contains several well-known pathogens but has also been found to form part of the microbiome of hematophagous invertebrates in the ocean [55]. Possibly, the *Vibrio* detected here could similarly be a part of the microbiome of deer keds, as it is seen in all RNA samples at a low level; further investigation is needed.

It must however be acknowledged that this study was limited by the number of deer keds sequenced, thus restricting the conclusions that can be drawn from these results, as does the fact that the samples from one location were all blood fed and the samples from the other were all unfed. Nevertheless, the results presented here provide a springboard for future studies that are both larger and more wide ranging in terms of sample numbers and geography and in the comparison of lifestages and effects of blood feeding.

## Conclusions

This study has determined the microbiome of UK deer keds through mass sequencing. This has identified a range of microorganisms that reside within these invertebrates and has identified several organisms of interest. We have identified, for the first time to our knowledge, a novel virus, *Sigmavirus lipoptenae*, infecting *L. cervi*; it is related to viruses that can infect mammals and are potential arboviruses. The work also showed that *L. cervi* in the UK carry the bacterial pathogen *B. schoenbuchensis*, which is known to cause disease in humans, and therefore shows that *L. cervi* could be a vector of zoonotic pathogens.

## Supplementary Information

Supplementary Material 1. Bayesian phylogeny of Hippoboscidae mitochondrial coding region. Phylogenies were created using MrBayes with 1,000,000 generations of Markov chain Monte Carlo simulations under the GTR + G + I model. The best model for each dataset was predicted using Modeltest-ng (v0.1.7.). Each node is labelled with Bayesian posterior probability. Sequences obtained in this study are highlighted in red. Phylogeny has been rooted on a *Glossina brevipalpis* isolate. Accession numbers for each of the six mitochondrial genomes from *Lipoptena cervi* samples in this study are PV090976–PV090981.

Supplementary Material 2. Bayesian phylogeny of Bartonella MLST markers. A 2703-bp sequence based on the concatenation of fragments of six genes (16S, ftsZ, gltA, nuoG, ribC, and rpoB). Sequences obtained in this study are highlighted in red. The remaining concatenated sequences were acquired from the supplementary material of Vogt et al. [38]. As sequences are concatenations of multiple sequences, there no accession numbers are available; therefore, strain ID, in brackets, has been provided. Phylogenies were created using MrBayes with 1,000,000 generations of Markov chain Monte Carlo simulations under the HKY + I model. The best model for each dataset was predicted using Modeltest-ng (v0.1.7.). Each node is labelled with Bayesian posterior probability. Phylogeny has been rooted on a *Bartonella dromedarii* isolate. Accession numbers for each of the six genes from samples in this study are PV155504–PV155506, PX206320–PX206331 and PX208520–PX208522.

Supplementary Material 3. Concatenated MLST sequences. An alignment in FASTA format of the concatenated sequences of the six MLST sequences for the isolates from this study and Vogt et al. [38].

## Data Availability

The datasets generated and/or analysed during the current study are available in the NCBI repository, accession nos. PV090976–PV090981, PV155500–PV155506, PV174796–PV174801, PX206320–PX206331 and PX208520–PX208522. Bioproject ID: PRJNA1246507.
