# Supplementary figures and images for: Detection of Bartonella schoenbuchensis and a novel sigmavirus within the microbiome of deer keds (Lipoptena cervi) from the United Kingdom
Source: Parasit Vectors. 2026 Jan 25;19:89. doi: 10.1186/s13071-025-07208-w (PMC12915030; doi:10.1186/s13071-025-07208-w)

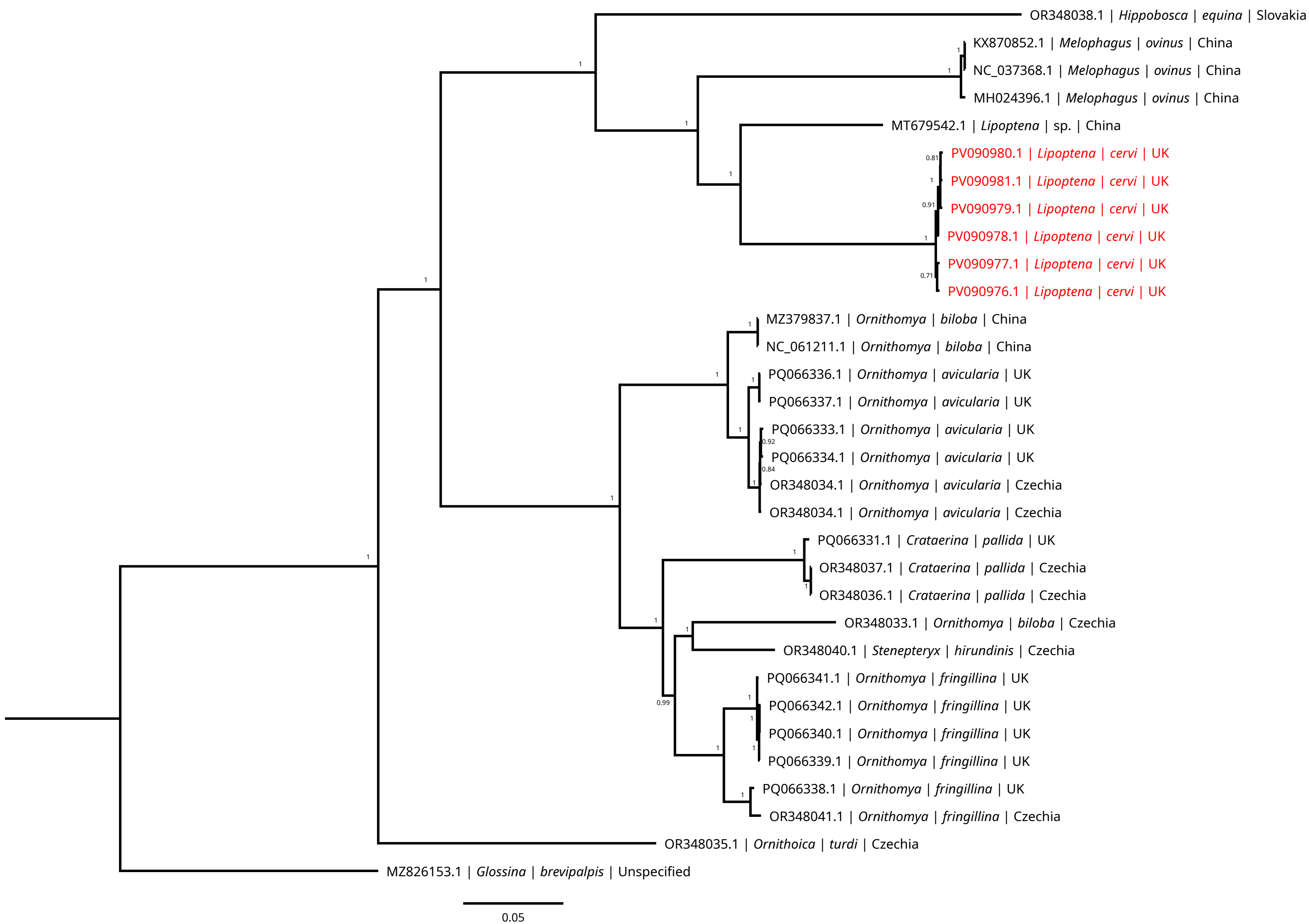

Supplement: Supplementary file 1 — Supplementary Material 1. Bayesian phylogeny of Hippoboscidae mitochondrial coding region. Phylogenies were created using MrBayes with 1,000,000 generations of Markov chain Monte Carlo simulations under the GTR + G + I model. The best model for each dataset was predicted using Modeltest-ng (v0.1.7.). Each node is labelled with Bayesian posterior probability. Sequences obtained in this study are highlighted in red. Phylogeny has been rooted on a Glossina brevipalpis isolate. Accession numbers for each of the six mitochondrial genomes from Lipoptena cervi samples in this study are PV090976–PV090981. [file 13071_2025_7208_MOESM1_ESM.pdf]

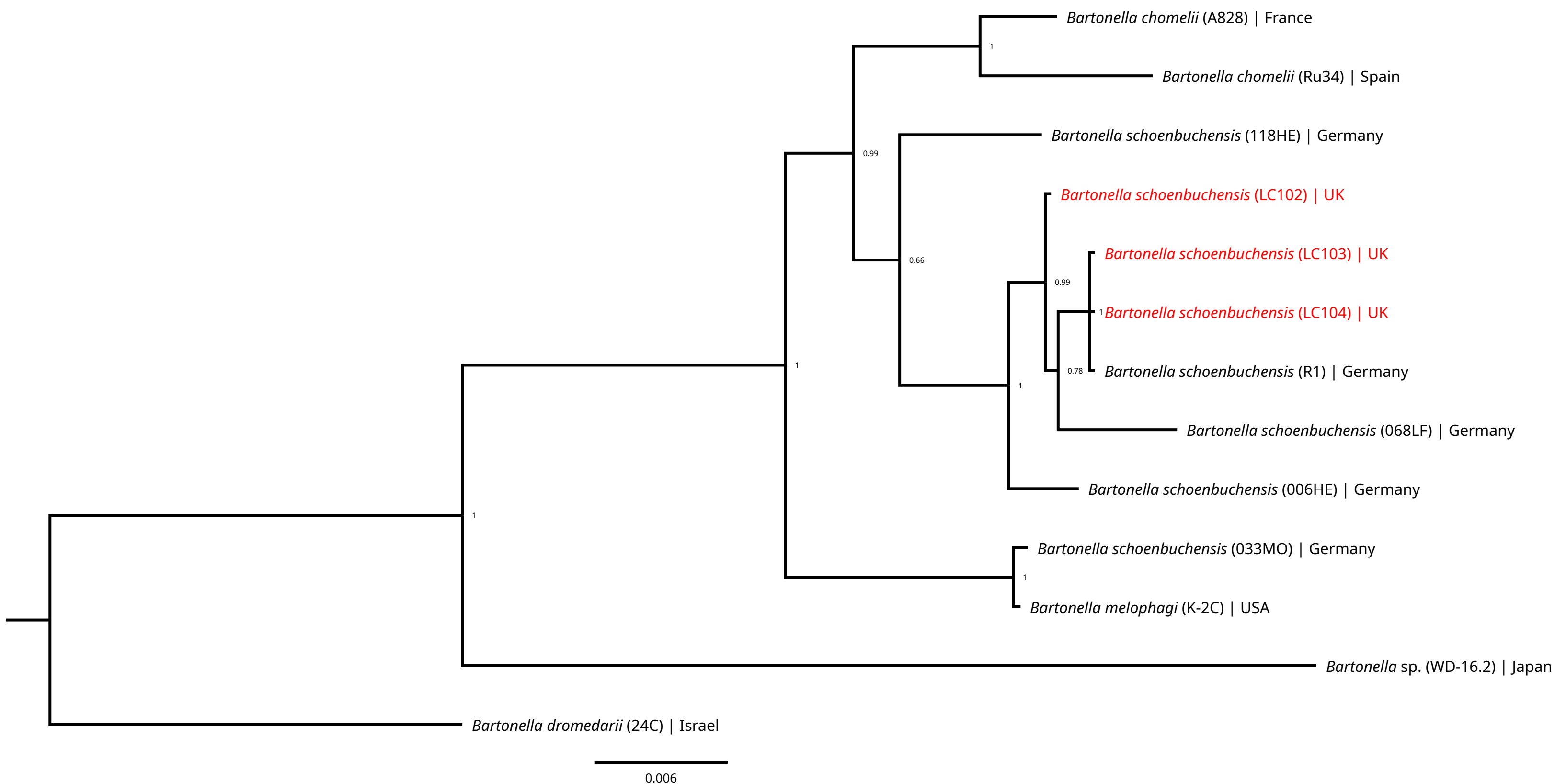

Supplement: Supplementary file 2 — Supplementary Material 2. Bayesian phylogeny of Bartonella MLST markers. A 2703-bp sequence based on the concatenation of fragments of six genes (16S, ftsZ, gltA, nuoG, ribC, and rpoB). Sequences obtained in this study are highlighted in red. The remaining concatenated sequences were acquired from the supplementary material of Vogt et al. [38]. As sequences are concatenations of multiple sequences, there no accession numbers are available; therefore, strain ID, in brackets, has been provided. Phylogenies were created using MrBayes with 1,000,000 generations of Markov chain Monte Carlo simulations under the HKY + I model. The best model for each dataset was predicted using Modeltest-ng (v0.1.7.). Each node is labelled with Bayesian posterior probability. Phylogeny has been rooted on a Bartonella dromedarii isolate. Accession numbers for each of the six genes from samples in this study are PV155504–PV155506, PX206320–PX206331 and PX208520–PX208522. [file 13071_2025_7208_MOESM2_ESM.pdf]
